# Idade Cronológica ou Idade Biológica, Principalmente uma Questão de Estilo de Vida

**DOI:** 10.36660/abc.20210732

**Published:** 2021-09-01

**Affiliations:** 1 Universidade Federal de Goiás Goiânia GO Brasil Universidade Federal de Goiás, Goiânia, GO - Brasil; 2 Universidade Federal de Goiás Goiânia GO Brasil Hospital do Coração de Goiás, Goiânia, GO - Brasil; 3 Brigham and Womens Hospital Harvard Medical School Boston Massachusetts Estado Unidos da América Harvard Medical School/Brigham and Womens Hospital, Boston, Massachusetts – Estado Unidos da América

**Keywords:** Idoso, Envelhecimento Saudável, Estilo de Vida, Rigidez Arterial, Índice de Massa Corporal, Obesidade, Estresse, Sedentarismo, Genética, Espiritualidade

Apesar das grandes disparidades socioeconômicas globais, a população mundial tem crescido de maneira exponencial e a vida média segue aumentando de maneira significativa.^1^^–^^4^

Se, no início do século 19, a expectativa de vida na Europa estava em torno dos 35 anos, na atualidade, apenas o continente africano tem populações com expectativa de vida média menor que 60 anos.^1^^–^^3^

Temos, em um extremo, o Japão, onde a vida média alcança 84 anos e no outro, Serra Leoa, na África, onde a expectativa é de 46 anos. No Brasil, em 2019, a expectativa de vida passou a 76,6 anos, sendo de 73,1 para homens e 80,1 anos para mulheres.^1^^–^^4^

Um conjunto de fatores determinou esta avassaladora modificação e, entre eles, destacam-se claramente as melhores condições de higiene, oferta de maior quantidade de alimentos, avanços na medicina e tecnologia e, dentro desta vertente, o desenvolvimento fantástico da imunização.

Com estas transformações, o conceito de idade passa a ser revisado, com o progressivo abandono da simples idade cronológica, para a agregação da moderna concepção de idade biológica.^4^^–^^6^

A própria convivência social e os costumes já se antecipam a esta realidade, reclassificando as faixas etárias, e no Brasil, a par dos considerados idosos por lei (> = 60 anos), Lei n.10.048, existem os que têm prioridade perante os demais idosos (> 80 anos), Lei n.13.466.

Com esta nova perspectiva, o estudo do envelhecimento vascular ganha cada vez mais importância, no sentido de identificar na população geral indivíduos que apresentem diferentes estágios de envelhecimento vascular e, consequentemente, diferentes riscos do aparecimento de doenças cardiovasculares, que são a principal causa de morte, modificando a história natural do indivíduo e encurtando sua vida útil.^4^^,^^7^^,^^8^

Os clássicos fatores de risco continuam a “comandar” o rol de possibilidades de desfechos circulatórios. Entretanto, o reconhecimento progressivo da associação e correlação destes, com o envelhecimento vascular, medido com mais precisão, começa a permitir uma atuação mais vigorosa para modificar o curso natural destas doenças.^4^^,^^9^^,^^10^^,^^11^

A composição corporal inadequada está incluída no rol dos fatores que interferem no risco cardiovascular e, neste particular, o artigo de Melo e Silva et al.,^12^ traz interessantes “insights”, é instigante e indica um caminho novo que vem sendo explorado mundialmente.

Trata-se de estudo transversal de uma coorte prospectiva de idosos com 80 anos ou mais, vivendo em comunidade, com independência funcional e cognitiva.

Foram estudados 124 longevos com idade média de 87,1 anos (74,5% mulheres e 57,3% brancos). A metodologia de investigação, tanto da composição corporal quanto da rigidez arterial, utilizou instrumentos precisos e validados, dando confiabilidade às medidas.

Foram correlacionados dados de rigidez arterial representados pela velocidade de onda de pulso (VOP), *augmentation index* (Aix), índice de amplificação da pressão de pulso (iAPP), pressão de pulso central (PPc), com os dados de composição corporal representados pela massa magra total (MM), a massa apendicular (MA), o percentual de gordura corporal e ainda o índice de Baumgartner (IB), calculado pelo equipamento que substitui a massa corporal total.

Os interessantes e provocativos achados mostraram correlação inversa significativa do AIx com a MM (r = −0,391, p<0,001), MA (r= −0,378, p< 0,001) e IB (r = −0,258, p 0,004). Além disso, a PPc apresentou associação inversa com MM (r= −0,268, p =0,003), MA (r = −0,288, p= 0,001) e IB (r= −0,265, p = 0,003). Houve relação direta apenas entre AIx e percentual de gordura corporal (r= 0,197, p= 0,029). Para a VOP não foram encontradas significâncias estatísticas.

O trabalho tem o mérito de ser o primeiro a avaliar a associação entre a rigidez arterial e composição corporal em idosos longevos na comunidade. Os resultados mostram que, quanto maior a quantidade de massa muscular, menor a rigidez arterial central e, ao contrário, quanto maior o percentual de gordura corporal, maior a rigidez arterial. Com relação à VOP, os achados foram semelhantes aos encontrados no estudo Partage (Predictive Values of Blood Pressure and Arterial Stiffness in Institutionalized Very Aged Population) que avaliou indivíduos com idades semelhantes, mais numeroso, com a diferença de serem institucionalizados.^13^

O encontro da fraca associação entre rigidez arterial e gordura corporal vai ao encontro da teoria do paradoxo da obesidade,^14^ o chamado viés da sobrevivência, ou seja, quem chegou à idade muito avançada, estaria livre dos malefícios metabólicos do excesso de gordura. Este fato, no entanto, não ameniza a diminuição da qualidade de vida, causada pela gordura corporal, pela perda da funcionalidade e o aumento da fragilidade.^15^

As limitações próprias de um estudo transversal, unicêntrico, com um número pequeno de indivíduos, não invalidam sua importância e mostram interessantes possibilidades futuras para estudos longitudinais, com amostras representativas que nos indicarão caminhos para a prevenção e tratamento precoce de agravos.

Este conceito moderno de envelhecimento, como propõem Sanderson e Scherbov, descreve que “a idade cronológica é a idade retrospectiva e mede quantos anos a pessoa já viveu. Todos da mesma idade viveram o mesmo número de anos. Em contraste, a idade prospectiva diz respeito ao futuro. Fala sobre os anos de vida a serem vividos. Os que tiverem a mesma idade prospectiva, estes sim, terão os mesmos anos de vida futura.”^5^^,^^6^

Este futuro está diretamente ligado à genética, mas, de maneira não menos importante, aos hábitos de vida, relações sociais, capacidade de lidar com estresse e o conceito mais amplo de espiritualidade. Um caminho novo que se descortina.
