# Supplementary material for: Idade Cronológica ou Idade Biológica, Principalmente uma Questão de Estilo de Vida
Source: Arq Bras Cardiol. 2021 Sep 1;117(3):463–4. [Article in Portuguese] doi: 10.36660/abc.20210732 (PMC8462961; doi:10.36660/abc.20210732)
Supplement: Translation

# Translation: Chronological Age or Biological Age, Mainly a Matter of Lifestyle

**Affiliations:** 1 Goiânia GO Brazil Universidade Federal de Goiás, Goiânia, GO - Brazil; 2 Goiânia GO Brazil Hospital do Coração de Goiás, Goiânia, GO - Brazil; 3 Boston Massachusetts United States of America Harvard Medical School/Brigham and Womens Hospital, Boston, Massachusetts - United States of America

**Keywords:** Aged, Healthy Aging, Life Style, Vascular Stiffness, Body Mass Index, Obesity, Stress, Sedentarism, Genetics, Spirituality

Despite the significant global socioeconomic disparities, the world's population has grown exponentially, and the average life continues to increase significantly.^1^^–^^4^

If at the beginning of the 19th century, life expectancy in Europe was around 35 years, at present, only the African continent has populations with an average life expectancy of fewer than 60 years.^1^^–^^3^

We have, at one extreme, Japan, where the average life reaches 84 years and the other, Sierra Leone, Africa, where the expectation is 46 years. In 2019, life expectancy in Brazil increased to 76.6 years, being 73.1 for men and 80.1 years for women.^1^^–^^4^

A set of factors determined this overwhelming change and, among them, the best hygiene conditions, the offer of a greater quantity of food, advances in medicine and technology and, within this aspect, the fantastic development of immunization stand out.

With these transformations, the concept of age begins to be revised, with the progressive abandonment of simple chronological age, to aggregate the modern conception of biological age.^4^^–^^6^

Social coexistence itself and customs are already anticipated in this reality, reclassifying age groups. In Brazil, along with those considered elderly by law (> = 60 years), Law no. 10,048, some have priority over the other elderly (> 80 years), Law no. 13,466.

With this new perspective, the study of vascular aging is gaining increasing importance to identify individuals in the general population who present different stages of vascular aging and, consequently, different risks of the onset of cardiovascular diseases, which are the main cause of death, modifying the individual's natural history and shortening its useful life.^4^^,^^7^^,^^8^

The classic risk factors continue to “command” the list of possibilities of circulatory outcomes. However, the progressive recognition of their association and correlation with vascular aging, measured more accurately, begins to allow a more vigorous action to modify the natural course of these diseases.^4^^,^^9^^,^^10^^,^^11^

Inadequate body composition is included in the list of factors that interfere with cardiovascular risk and, in this regard, the article by Melo e Silva et al.,^12^ brings interesting insights, is thought-provoking and indicates a new path that is being explored worldwide.

This is a cross-sectional study of a prospective cohort of older people aged 80 years or older living in a functional and cognitive independence community.

124 long-lived with a mean age of 87.1 years (74.5% women and 57.3% white) were studied. The investigation methodology used precise and validated instruments, both of body composition and arterial stiffness, giving reliability to the measurements.

Data on arterial stiffness represented by pulse wave velocity (PWV), augmentation index (Aix), pulse pressure amplification index (PPAi), central pulse pressure (cPP), with body composition data represented by total lean mass (LM), appendicular mass (AM), body fat percentage and Baumgartner index (BI), calculated by the equipment that replaces total body mass, were correlated.

The interesting and provocative findings showed a significant inverse correlation between AIx and LM (r = −0.391, p < 0.001), AM (r = −0.378, p < 0.001) and BI (r = −0.258, p 0.004). In addition, CPP presented an inverse association with LM (r = −0.268, p = 0.003), AM (r = −0.288, p = 0.001) and BI (r = −0.265, p = 0.003). There was a direct relationship only between AIx and body fat percentage (r = 0.197, p = 0.029). Statistical significance was not found for PWV.

The work has the merit of being the first to assess the association between arterial stiffness and body composition in long-lived older adults in the community. The results show that the greater the amount of muscle mass, the lower the central arterial stiffness and, on the contrary, the higher the percentage of body fat, the greater the arterial stiffness. Regarding PWV, the findings were similar to those found in the Partage (Predictive Values of Blood Pressure and Arterial Stiffness in Institutionalized Very Population Aged) study, which evaluated individuals of similar ages, more numerous, with the difference of being institutionalized.^13^

The encounter of the weak association between arterial stiffness and body fat is in line with the theory of the obesity paradox,^14^ the so-called survival bias, that is, those who reached a very advanced age would be free of the metabolic harms of excess fat. This fact, however, does not mitigate the decrease in quality of life caused by body fat, loss of functionality and increased frailty.^15^

The limitations of a cross-sectional, single-center study with a small number of individuals do not invalidate its importance and show interesting future possibilities for longitudinal studies, with representative samples that will indicate ways to prevent and early treat of injuries.

As proposed by Sanderson and Scherbov, this modern concept of aging describes that “chronological age is the retrospective age and measures how many years the person has lived. Every one of the same age lived the same number of years. In contrast, prospective age concerns the future. It talks about the years of life to be lived. Those who have the same prospective age, these yes, will have the same years of future life.”^5^^,^^6^

This future is directly linked to genetics, but, in no less significant way, to life habits, social relationships, the ability to deal with stress and the broader concept of spirituality. A new path that unfolds.
